# Subcutaneous IL-6 Inhibitor Sarilumab vs. Standard Care in Hospitalized Patients With Moderate-To-Severe COVID-19: An Open Label Randomized Clinical Trial

**DOI:** 10.3389/fmed.2022.819621

**Published:** 2022-02-23

**Authors:** Rosario García-Vicuña, Sebastián C. Rodriguez-García, Francisco Abad-Santos, Azucena Bautista Hernández, Lucio García-Fraile, Ana Barrios Blandino, Angela Gutiérrez Liarte, Tamara Alonso-Pérez, Laura Cardeñoso, Aránzazu Alfranca, Gina Mejía-Abril, Jesús Sanz Sanz, Isidoro González-Alvaro

**Affiliations:** ^1^Rheumatology Service, Instituto de Investigación Sanitaria Princesa (IIS-Princesa), Hospital Universitario La Princesa, Madrid, Spain; ^2^Faculty of Medicine, Universidad Autónoma de Madrid, Madrid, Spain; ^3^Department of Clinical Pharmacology, Clinical Research and Clinical Trials Unit (CRCTU), Instituto de Investigación Sanitaria Princesa (IIS-Princesa), Hospital Universitario La Princesa, Madrid, Spain; ^4^Division of Infectious Diseases, Internal Medicine Service, Instituto de Investigación Sanitaria Princesa (IIS-Princesa), Hospital Universitario La Princesa, Madrid, Spain; ^5^Pneumology Service, Instituto de Investigación Sanitaria Princesa (IIS-Princesa), Hospital Universitario La Princesa, Madrid, Spain; ^6^Department of Microbiology, Instituto de Investigación Sanitaria Princesa (IIS-Princesa), Hospital Universitario La Princesa, Madrid, Spain; ^7^Immunology Service, Instituto de Investigación Sanitaria Princesa (IIS-Princesa), Hospital Universitario La Princesa, Madrid, Spain

**Keywords:** COVID-19, sarilumab, subcutaneous route, IL-6, IL-6 receptor inhibitors, IL-6 blockade, randomized controlled trial, subcutaneous

## Abstract

**Background:**

The use of IL-6 blockers in COVID-19 hospitalized patients has been associated with a reduction in mortality compared to standard care. However, many uncertainties remain pertaining to optimal intervention time, administration schedule, and predictors of response. To date, data on the use of subcutaneous sarilumab is limited and no randomized trial results are available.

**Methods:**

Open label randomized controlled trial at a single center in Spain. We included adult patients admitted with microbiology documented COVID-19 infection, imaging confirmed pneumonia, fever and/or laboratory evidence of inflammatory phenotype, and no need for invasive ventilation. Participants were randomly assigned to receive sarilumab, a single 400 mg dose in two 200 mg subcutaneous injections, added to standard care or standard care, in a 2:1 proportion. Primary endpoints included 30-day mortality, mean change in clinical status at day 7 scored in a 7-category ordinal scale ranging from death (category 1) to discharge (category 7), and duration of hospitalization. The primary efficacy analysis was conducted on the intention-to-treat population.

**Results:**

A total of 30 patients underwent randomization: 20 to sarilumab and 10 to standard care. Most patients were male (20/30, 67%) with a median (interquartile range) age of 61.5 years (56–72). At day 30, 2/20 (10%) patients died in the sarilumab arm vs. none (0/10) in standard care (Log HR 15.11, SE 22.64; *p* = 0.54). At day 7, no significant differences were observed in the median change in clinical status (2 [0–3]) vs. 3 [0–3], *p* = 0.32). Median time to discharge (days) was similar (7 [6–11] vs. 6 [4–12]; HR 0.65, SE 0.26; *p* = 0.27). No significant differences were detected in the rate of progression to invasive and noninvasive mechanical ventilation.

**Conclusions and Relevance:**

Our pragmatic pilot study has failed to demonstrate the benefit of adding subcutaneous sarilumab to standard care for mortality by 30 days, functional status at day 7, or hospital stay. Findings herein do not exclude a potential effect of sarilumab in severe COVID-19 but adequately powered blinded randomized phase III trials are warranted to assess the impact of the subcutaneous route and a more selected target population.

**Trial Registration:**

www.ClinicalTrials.gov, Identifier: NCT04357808.

## Introduction

Approaching the second year after WHO declared COVID-19 as a pandemic, many uncertainties persisted about the disease course, prognosis, and treatment. Vaccination has emerged as the real hope for the global threat, but global herd immunity will take months or even years to be reached (1). Therefore, thousands of patients will still require supportive and pharmacological treatment.

During the early days of the pandemic, the rapid spread of the SARS-CoV-2 coronavirus posed unprecedented challenges for health services to properly manage COVID-19 severe and critical manifestations affecting a wide population in a short period of time. Given the ineffective antiviral therapy on hospitalized patients (2), huge efforts were directed to abrogate the hyperinflammatory status that complicates the clinical course and eventually leads to death (3). Off-label use of plenty of immunomodulatory drugs emerged, targeting cytokines involved in COVID-19 acute respiratory distress syndrome (ARDS), where high IL-6 levels have a prominent role (4). Tocilizumab (TCZ), an IL-6 receptor (R) inhibitor, was the first anti-cytokine agent tested in the pandemic (5–7), based on the pathogenic role of IL-6 as a driver of hyperinflammation (4, 8) and high IL-6 levels as predictors of poor outcomes (9–11). Consistent with those results, an observational study conducted in our hospital during the first outbreak in Spain demonstrated that early IL-6 blockade with TCZ was associated with improvement of oxygenation and reduced the death rate in patients with IL-6>30 pg/ml, as this was the best predictor of invasive mechanical ventilation (IMV) (12). In those early days, intravenous IL-6R inhibitors began to be tested in several trials; however, no data on subcutaneous formulations were available.

Sarilumab (SAR) is a human monoclonal antibody that binds membrane-bound and soluble IL-6 receptors to inhibit IL-6 signaling, licensed in a subcutaneous route administration for the treatment of Rheumatoid Arthritis (13). At a moment where the health system was overrun, especially emergency and intensive care unit (ICU) facilities, with real concern about TCZ shortages, we conceived that subcutaneous administration of SAR could facilitate the administration of an IL-6 inhibitor in all settings, including wards and overloaded emergency rooms. Additionally, the safety and maximum pharmacodynamic effects of a single 200 mg dose of subcutaneous SAR are known through the results of two open randomized controlled trials (RCT) (14). Data were similar to those obtained with single doses of 4 and 8 mg/kg intravenous TCZ, with a longer effect of TCZ in the second week. Our hypothesis was that the use of 2 subcutaneous SAR injections and early intervention (window of opportunity) could prevent higher oxygenation requirements through non-invasive (NI) and invasive mechanical ventilation (IMV) and reduce death rate. Thus, we proposed an open pilot pragmatic RCT to evaluate the efficacy and safety of a single 400 mg subcutaneous dose of SAR, in patients with moderate to early severe COVID-19, compared to standard care (SC).

## Methods

### Design

SARCOVID is an investigator-initiated open-label phase II RCT, conducted from April 13 (first patient enrolment) to December 4 (last patient's last visit), 2020, at Hospital Universitario La Princesa (HUP) during the first and second outbreak in Madrid, Spain. This design was a counterproposal from the Spanish Agency for Medicines and Health products (AEMPS) to our urgent proposal of an exploratory propensity score-matched observational study. The trial was approved by the AEMPS and the Research Ethics Committee of the HUP on April 9, 2020 (Reference number 4078) and was conducted in accordance with the principles of the Declaration of Helsinki and the Good Clinical Practice guidelines of the International Conference on Harmonization.

The timeline of recruitment is illustrated in Supplementary Figure 1. Enrolment abruptly dropped following the decrease of COVID-19 incidence in Madrid. A formal amendment was submitted to the HUP Ethics Committee on May 7, 2020, for the inclusion of a positive serologic test (IgM/IgA by ELISA) as diagnostic confirmation of COVID-19 infection in the absence of a positive reverse-transcriptase–PCR (RT-PCR) assay for SARS-CoV2 in a respiratory tract specimen. After concomitant approval of the AEMPS, trial recruitment remained open until completion. A full version of the protocol and amendment, which includes the statistical plan, has been published elsewhere (15). This study followed the Consolidated Standards of Reporting Trials (CONSORT) reporting guideline (See Supplementary Material).

### Study Population

Patients ≥18 and <80 years attending the emergency room of HUP in need for hospitalization or those in hospital wards were eligible if they had confirmed pneumonia on chest imaging and a documented diagnosis of COVID-19 by RT-PCR assay or, in its absence, case definition of COVID-19 pneumonia as per local protocol and a positive IgM/IgA serologic ELISA test. For recruitment, at least 2 of the following additional criteria needed to be fulfilled: Fever ≥ 37.8°C; IL-6 in serum ≥ 25 pg/mL or PCR > 5 mg / dL; lymphocytes <600/mm^3^; ferritin > 300 μg /L doubling in 24 h; ferritin > 600 μg /L in the first determination; and LDH > 250 or D-dimer > 1 mg / L. Exclusion criteria included requirements of IMV at inclusion; AST / ALT values more than 5-folds the upper normal limit; absolute neutrophil count <500/ mm^3^; absolute platelet count <50,000/ mm^3^; superimposed infection by pathogens other than COVID-19; complicated diverticulitis or intestinal perforation; immunosuppressive anti-rejection therapy; pregnancy or lactation; previous treatment with TCZ or SAR; contraindication to SAR or excipients; and comorbidities that can likely lead to an unfavorable result.

### Randomization and Treatments

A total of 30 patients were randomly allocated in a 2:1 ratio to the intervention group, SAR (400 mg single dose in 2 subcutaneous injections 200 mg each in pre-filled syringe) plus SC, or current SC. Central telephone randomization was performed by the Clinical Research and Clinical Trials Unit (CRCTU) at the HUP using the program www.randomization.com with a 2:1 proportion and 5 blocks of 6 subjects. After checking that all entry criteria were met, the CRCTU communicated the assigned treatment to the recruiting investigator, who reported the correct allocation in the electronic clinical record (ECR). Patients in both arms received drugs, including corticosteroids, or full supportive care according to the best SC updated in the local protocol for COVID-19. Patients in the SC were given the option to receive intravenous TCZ after randomization if they worsened at the investigator's discretion, as this agent had become the SC in our center when the protocol was designed (12). Other immunomodulators or investigational drugs in trials were prohibited.

### Outcomes

The primary endpoints were mortality by 30 days, mean change in functional status at day 7 on a 7-category ordinal scale as recommended by the WHO R&D Blueprint Group (16) (1. death; 2. hospitalized, requiring ECMO, IMV, or both; 3. hospitalized, requiring high-flow oxygen therapy, NIMV, or both; 4. hospitalized, requiring supplementary oxygen; 5. hospitalized, not requiring supplementary oxygen but in need of ongoing medical care; 6. hospitalized, not requiring ongoing medical care; and 7. not hospitalized), and time to discharge from randomization. Secondary outcomes included time to become afebrile during 48 h without antipyretics, mean change in 7-category ordinal scale at day 14, time to NIMV and IMV, time to oxygen supply independence, and adverse events (AE). As no events occurred in SC, the outcomes time to NIMV and IMV were changed to progression to NIMV and IMV.

As this trial has been included in a recent prospective meta-analysis of IL-6 inhibitors in hospitalized COVID-19 patients (17), mortality by 90 days and serious infections by 90 days were also assessed, although these outcomes were not included in the original protocol.

### Procedures

This trial was intended to be carried out pragmatically according to the usual clinical practice in Spain during the first pandemic wave, avoiding any additional workload in treating physicians who assessed each patient several times a day. The study calendar and procedures are detailed in the protocol. Briefly, vital signs, targeted physical examination, supplementary oxygen requirements, and resting oxygen saturation were recorded daily and registered at admission and subsequent study visits at days 0, 1, 2, 5, 7, and 14 after randomization, and at discharge day. Efficacy assessments included an evaluation of clinical status by a 7-category ordinal scale at days 0 (randomization), 7, and 14 (through a phone call if the patient had been discharged).

Laboratory testing was performed locally according to clinical practice at established study visits when the patient continued to be at the hospital. IL-6 serum levels were determined at baseline, day 5, and on patients still admitted at day 14. Serum IL-6 was quantified in duplicate with the Human IL-6 Quantikine high-sensitivity ELISA from R&D Systems Europe Ltd (Abingdon, United Kingdom), following the manufacturer's instructions.

On day 30 after randomization and days 10–15 after discharge, the patient appraisal was performed through a phone call by a member of the research team. Screening for tuberculosis, Hepatitis B Virus, and HIV was also done on day 0, and safety and concomitant medication assessments were performed daily until discharge. Although not included in the protocol, a review of the ECR, including microbiological isolations and drug prescriptions, both in hospital and primary care settings, was done to assess mortality and infections by 90 days. Patients with no recorded data for this timeframe were interviewed through a phone call by the principal investigator.

All patient data were anonymized and recorded into a local database.

### Data Quality Monitoring

Data quality on-site monitoring was performed by a dedicated staff of the CRCTU at the HUP, independent of the investigators' team, with 100% source data verification for all critical data points. All severe AE (SAE) was reviewed and evaluated by a qualified pharmacovigilance expert of the CRCTU, independent of the investigators' team.

### Statistical Analysis

A formal calculation of the sample size was not performed, since the study was designed as a pragmatic proof of concept study with a drug that had not been previously evaluated in COVID-19. Regulatory authorities (AEMPS) estimated that 30 patients (20 SAR: 10 control) might be sufficient for an initial evaluation of the study objectives.

Qualitative variables were described using a calculation of the proportions and due to the low number of patients in the SC group, the two-sided Fisher's exact test was used to compare categorical variables. Quantitative variables were represented as median and IQR and, considering the sample size, Mann–Whitney ***U***test was used to analyze significant differences. Statistical significance was considered if *p* < 0.05.

The primary efficacy analysis was conducted on the intention-to-treat (ITT) population. To estimate the intervention effect size, hazard ratio (HR) was estimated when it was feasible. In these cases, follow-up was censored to 30 days, which was the longest duration of hospitalization since randomization. For the total length of hospital stay, follow-up was censored to 33 days. For HR estimation, dead patients were assigned 30- or 33-day follow-ups, respectively.

As three patients received TCZ in the SC group, sensitivity analyses for primary and secondary outcomes were performed excluding those patients.

To provide a more accurate assessment of our results, avoiding biases, we performed a multivariable linear regression using generalized linear models (*glm* command of Stata) with the primary outcome change in clinical status on the 7-category ordinal scale at day 7 as the dependent variable, and several independent variables that could be confounding factors (age, gender, baseline category in the ordinal scale, time from symptoms onset, comorbidities, cumulative glucocorticoid use …among others). Since there were 30 cases, we first tested the independent variables one by one, and then with those with a better performance we fitted the best model with 3 independent variables, namely, gender, cumulative glucocorticoid dose between baseline and day 7, and the allocated treatments.

Statistical analyses were performed using Stata 14.0 for Windows (Stata Corp LP, College Station, TX, United States).

## Results

### Patients

**Figure 1:**
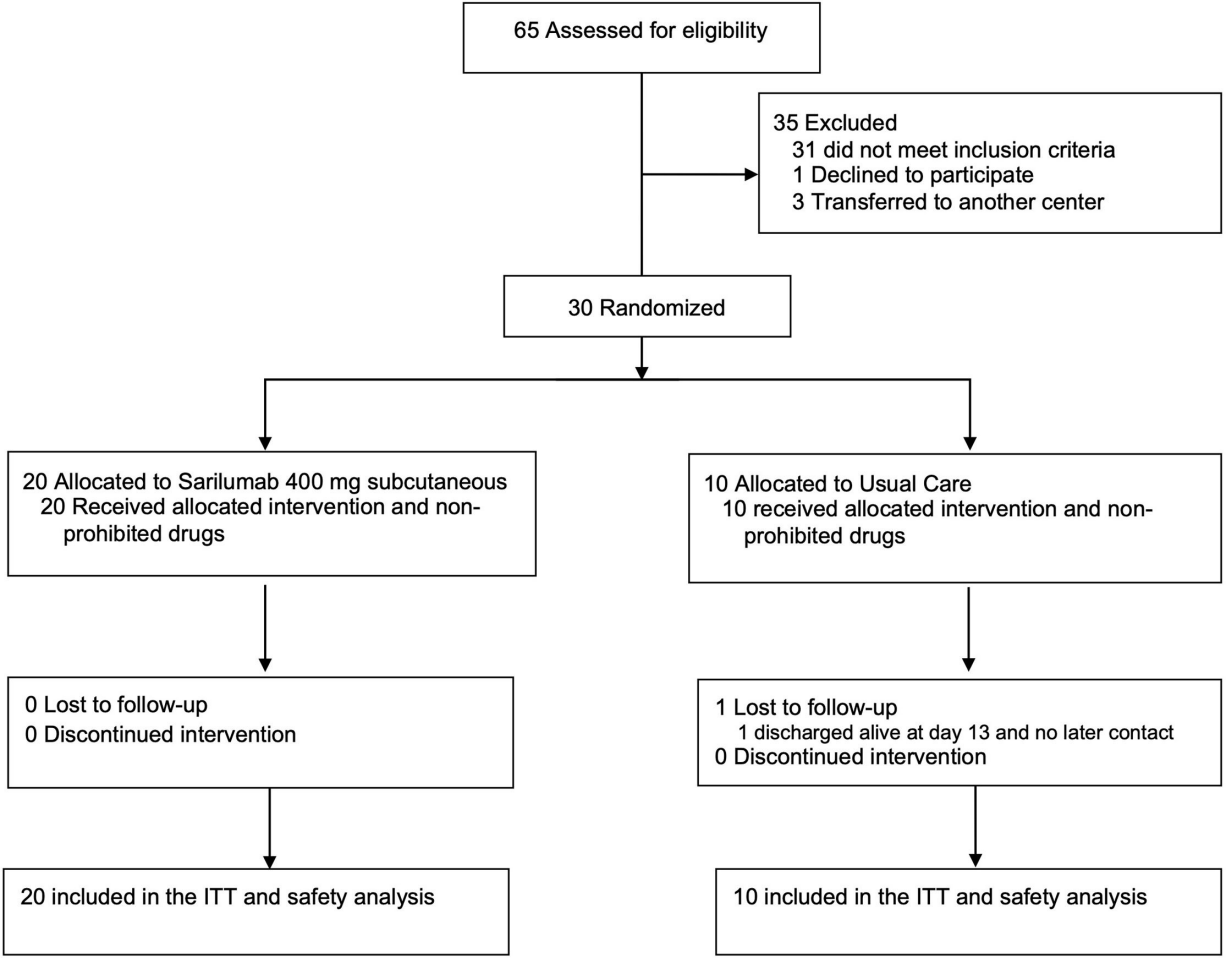
Flow chart of the study.

Between April 13 and October 30, 2020, 30 of 65 screened patients underwent 2:1 randomization: 20 to SAR (400 mg single dose, subcutaneous) + SC and 10 to SC. The last patient's last visit was on December 4, 2020. All patients received the allocated intervention and completed the follow-up, except 1 in the SC arm, who was discharged alive on day 13, with no response to the study's post-discharge appointment (Figure 1). Of the 10 patients assigned to SC, 3 received late TCZ after randomization and were included in the ITT analysis on the arm they were originally allocated. The median age was 61.5 years (IQR 56**–**72), 67% were men (Table 1). All randomized patients had finally a documented diagnosis of COVID-19 by RT-PCR assay. Most clinical, demographic, and laboratory baseline data (Tables 1, 2, respectively) were similar across treatment groups. However, a higher proportion of men, fever, and extended radiological pattern at admission was recorded in the SAR arm, along with a shorter duration from symptom onset to randomization. All patients receiving high flow oxygen therapy or NIMV at randomization were allocated to SAR while all patients under SC had low flow supplementary oxygen requirements. Conversely, 4/20 (20%) in the SAR arm had no supplementary oxygen requirements.

**Table 1:** Baseline demographics and clinical characteristics of the study population.

	**n (%)**		
	**Total (*n* = 30)**	**Sarilumab + SC (*n* = 20)**	**SC (*n* = 10)**
**Median Age in years (IQR)**	61.5 (56–72)	61.5 (50.5–72)	62 (58–71)
**Male sex, n (%)**	20 (67)	15 (75)	5 (50)
**Race, ethnicity (%)**			
White	14 (47)	10 (50)	4 (40)
Asian	1 (3)	0 (0)	1 (10)
Hispanic or latino	15 (50)	10 (50)	5 (50)
**Coexisting Disorders, n (%)**	19 (63)	14 (70)	5(50)
Hypertension	13(43)	8 (40)	5 (50)
Diabetes Mellitus	5 (17)	3 (15)	2 (20)
Obesity	3 (10)	2 (10)	1 (10)
History of Malignancy	2 (7)	2 (10)	0 (0)
COPD	2 (7)	1 (5)	1 (10)
Stage III Chronic kidney disease	4 (13)	2 (10)	2 (20)
Coronary artery disease	3 (10)	3 (15)	0
**Median days from symptom onset to randomization (IQR)**	11 (8-16)	10.5 (8-12.5)	16 (12-23)
**Median days from admission to randomization (IQR)**	2 (1-4)	2 (1-4)	3 (1-6)
**Median body temperature at randomization (IQR)**, **°C**	37 (36.4-37.7)	37.1 (36.6-38.1)	36.5 (36.3-37.2)
**Fever** **≥37,5°C, n (%)**	10 (33)	9 (45)	1 (10)
**Oxygen support at randomization (7-category ordinal scale) n (%)**			
5. No supplemental oxygen therapy	4 (13.3)	4 (20)	0 (0)
4. Supplemental low flow oxygen therapy^a^	22 (73.3)	12 (60)	10 (100)
3. High-flow supplemental oxygen therapy or NIV^b^	4 (13.3)	4 (20)	0 (0)
Median PaO_2_/FiO_2_ mmHg (IQR) at randomization	318 (233-358)	298 (223-348)	341 (261-404)
**Additional treatment during hospitalization**			
Hydroxychloroquine	6 (20)	4 (20)	2 (20)
Lopinavir/Ritonavir	5 (17)	4 (20)	1 (10)
Azithromycin	18 (60)	12 (60)	6 (60)
Interferon	0 (0)	0 (0)	0 (0)
Remdesivir at randomization	0 (0)	0 (0)	0 (0)
Corticosteroids at randomization^c^	25 (83)	17 (85)	8 (80)
Methylprednisolone bolus	17 (57)	14 (70)	3 (30)

*COPD, Chronic obstructive pulmonary disease; IQR, interquartile range; NIV, noninvasive ventilation; PaO_2_/Fi0_2_, partial pressure of arterial oxygen/fraction of inspired oxygen; SC, standard care*.

a *O_2_ flow ≤ 15 l/min e.g., by face mask, nasal cannula (NC)*.

b *O_2_ flow >15 l/min, e.g., by face mask, ‘High Flow' devices (e.g., HFNC), CPAP or NIV including BiPAP and other devices*.

c *Corticosteroids: ≥ 30 mg Prednisone/d or equivalent; endovenous bolus of 6-Metilprednisolone 120–125 mg/d, except for 1 patient 80 mg/dl*.

**Table 2:** Baseline laboratory and radiologic findings of the study population.

	**n (%)**		
	**Total (*n* = 30)**	**Sarilumab + SC (*n* = 20)**	**SC (*n* = 10)**
**Laboratory values (median, IQR)**			
White Blood Count (cells/mm^3^)	7,985 (5,160–11,140)	7,070 (4,975–12,310)	10,065 (6,750–10,460)
Lymphocyte Count (cells/mm^3^)	830 (680–1,130)	825 (680–1,070)	865 (680–1,580)
Neutrophil Count (cells/mm^3^)	5,910 (3,935–9,312)	6,215 (5,398–9,313)	5,850 (3,575–10,972)
Creatinine. mg/dL	0.80 (0.63–0.98)	0.83 (0.71–0.99)	0.65 (0.59–0.87)
Bilirubin. mg/dL	0.40 (0.32–0.52)	0.38 (0.32–0.53)	0.49 (0.34–0.52)
AST. U/L	33 (26-54)	40 (27-53)	32 (25-93)
ALT. U/L	46 (24-61)	48 (29-57)	28 (21-97)
GGT. U/L	56 (34-117)	41 (30-119)	71 (55–108)
LDH. U/L	297 (238–349)	317 (263–350)	263 (222–333)
**Inflammatory markers**			
serum IL-6. pg/mL (*n* = 24)	12 (3–21.5)	13.3 (7.5–24)	3 (1–16.5)
IL-6 ≥ 30 pg/mL, n (%)	4/24 (17)	3/16 (19)	1/8 (13)
Ferritin. ng/mL (*n* = 29)	1,179 (735–1,511)	1,048 (664–1,511)	1,265 (735–1,532)
CRP. mg/dL	9.28 (5.06–19.73)	8.59 (4.17–18.1)	9.94 (6.19–19.73)
PCT. ng/mL (*n* = 13)	0.11 (0.08–0.18)	0.11 (0.09–0.18)	0.12 (0.07–0.18)
D-dimer (μg/mL) (*n* = 29)	0.49 (0.37–1.14)	0.49 (0.36–1.28)	0.51 (0.37–1.09)
**Baseline thorax radiologic findings (x ray and/or CT scan)** ^a^			
Alveolar pattern or ground glass opacities > 50%	14 (47)	11 (55)	3 (30)

*AST, Aspartate amino-transferase; ALT, Alanine amino- transferase; CRP, C-reactive protein; GGT, Gamma-glutamyl transferase; IL-6, Interleukin-6; IQR, Interquartile range; LDH, Lactate Dehydrogenase; PCT, Procalcitonin. SC, Standard care*.

a *All radiologic exams were assessed and reported by radiologists with pneumological expertise*.

Median CRP levels were 9.28 (IQR 5.06**–**19.73) mg/dl without significant differences between allocations at randomization (Table 2). Serum IL-6 levels were available in 24 patients at randomization, with a median of 13.3 (IQR 7.5**–**24) pg/ml on 16 SAR patients and 3 (IQR 1**–**16.5) pg/ml on 8 SC subjects. Only 3/16 patients in the intervention arm and 1/8 in SC had high IL-6 levels (Table 2), according to the threshold (30 pg/ml) previously established in our hospital population (12).

More than 80% of patients received glucocorticoids at randomization (Table 1), with no significant differences between arms in the median accumulated dose before randomization (156 mg [IQR 90**–**300] in SAR vs. 207 mg [IQR 80**–**550] in SC, ***p*** = 0.81) and after allocation (105 mg [IQR 0**–**403] in SAR vs. 135 mg [100**–**200] in SC, ***p*** = 0.80).

### Primary Outcomes

**Figure 2:**
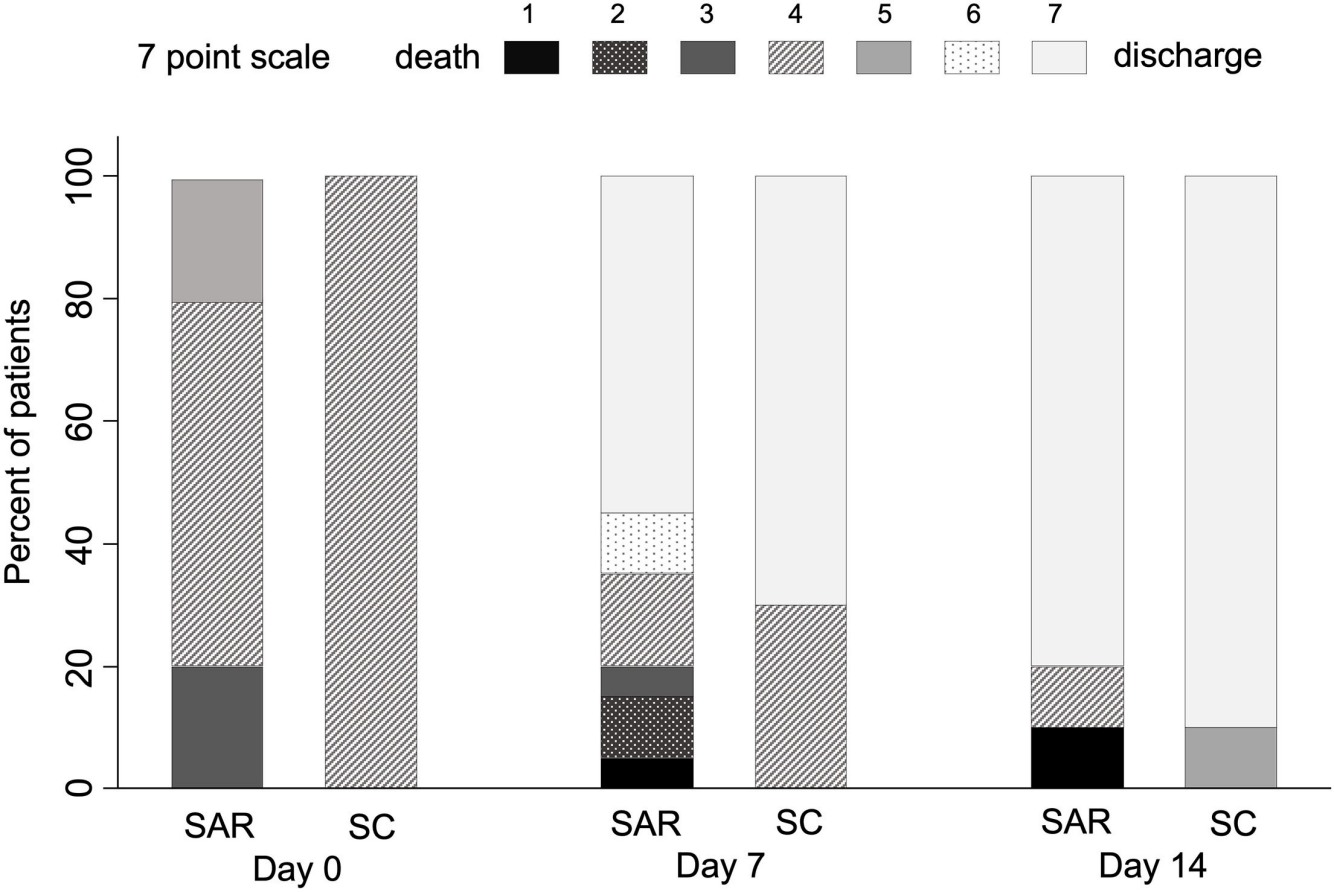
Evolution of clinical status in COVID-19 patients from baseline to day 14 according to the 7-category ordinal scale. Data are shown as the percentage of patients at each ordinal point in the sarilumab + standard care (SAR; *n* = 20) and standard care (SC; *n* = 10) groups, displayed as boxes with the different hues ranging from black (1 = death) to white (7 = discharge) scale.

No significant differences were seen in the median change [IQR] in clinical status on the 7-category ordinal scale at day 7 between SAR and SC (2 [0**–**3] vs. 3 [0**–**3], ***p*** = 0.32) (Table 3). The proportion of patients in each category at this time point is shown in Figure 2. Regarding 30-day mortality, 2/20 (10%) patients died in the SAR arm while no events (0/10) were found in SC (Table 3). Those results were identical for in-hospital mortality. Two deaths occurred in patients with previous Grade III chronic kidney disease (CKD) and NIMV at randomization. Median days to discharge on SAR and SC were similar (HR = 0.65, SD = 0.26; *p* = 0.27).

**Table 3:** Clinical outcomes in the intention-to-treat population.

	**Median (IQR)**				
**Outcomes**	**Sarilumab + SC (*n* = 20)**	**SC (*n* = 10)**	**Hazard ratio (SE)**	**Log hazard ratio**	***P*** **-Value**
				**(Log SE)**	
**Primary outcomes**					
Median change in clinical status (7-category ordinal scale ^a^) at day 7,	2 (0–3)	3 (0–3)			0.32
Mean change on clinical status at day 7, (SD)	1.45 (1.93)	2.1 (1.45)			0.36
30-day mortality, n (%) ^b^	2 (10)	0		15.11 (22.64)	0.54
Duration of hospitalization, days from randomization ^c^	7 (6-11)	6 (4-12)	0.65 (0.26)		0.27
**Secondary outcomes**					
Median change in clinical status at day 14	3 (3)	4 (2-4)			0.36
Time to become afebrile for a minimum of 48 h, days^d^	3 (3-6)	4 (4-8)	1.60 (0.97)		0.39
Progression to NIMV, n (%)	4 (20)	0 (0)		15.09 (22.52)	0.27
Progression to IMV, n (%)	3 (15)	0 (0)		15.10 (22.52)	0.5
Time to supplemental oxygen withdrawal, days from randomization	5.5 (3−13)	4.5 (2-12)	0.83 (0.37)		0.83

*IMV, invasive mechanical ventilation; IQR, interquartile ranges; NIMV, Noninvasive mechanical ventilation; SD, standard deviation: SC, Standard care; SE, Standard error*.

a *Scale range: 1 = death to 7 = non hospitalized*.

b *One patient in the SC arm was lost to follow-up after discharge at day 13*.

c *Accounting for survival status by treating patients who died as having a 30-day hospital stay*.

d *Eleven patients in the SAR+SC arm and 5 in the SC arm were febrile at randomization*.

We performed a multivariate analysis to determine which variables influenced the primary outcome “change in clinical status at day 7” that ranges from death (0) to discharge (7). Along with other confounding factors, age was not significantly associated with this outcome in the bivariate analysis and finally, the best multivariate model included 3 independent variables: sex/gender, cumulative glucocorticoid dose between baseline and day 7, and the allocated treatments. The results (Table 4) showed that higher requirements of glucocorticoids after randomization were significantly associated with a worse clinical evolution at day 7, likely reflecting a confounding by indication bias, as those patients that rapidly worsened were prescribed higher doses of corticosteroids. In addition, the female gender showed a trend to worse evolution. After adjustment by these variables, there were no significant differences between SC and SC plus SAR.

**Table 4:** Variables associated with improvement in clinical status at day 7.

	**β Coefficient**	**95% Confidence interval**	***p*** **-value**
**Treatment**			
SC	Reference	-	0.156
SC + Sarilumab	−0.89	−2.11 – 0.34	
**Gender**			
Male	Reference	-	0.085
Female	−1.07	−2.30 – 0.15	
**Cumulative GC dose (by g of prednisone)** ^a^	−1.92	−3.27 to −0.58	0.005

*SC, standard care; GC, glucocorticoids; g, gram*.

a *Cumulative GC dose from randomization to day 7*.

### Secondary Outcomes

No significant differences were observed between arms for any of the secondary outcomes (Table 3). In SAR, 4/20 (20%) and 3/20 (15%) patients required NIMV and IMV, respectively, vs. none in the SC. Notably, 2/3 of patients progressing to IMV were not receiving corticosteroids at randomization day, although just one of these 2 patients died. The median time to oxygen withdrawal was similar between groups (Table 3).

**Figure 3:**
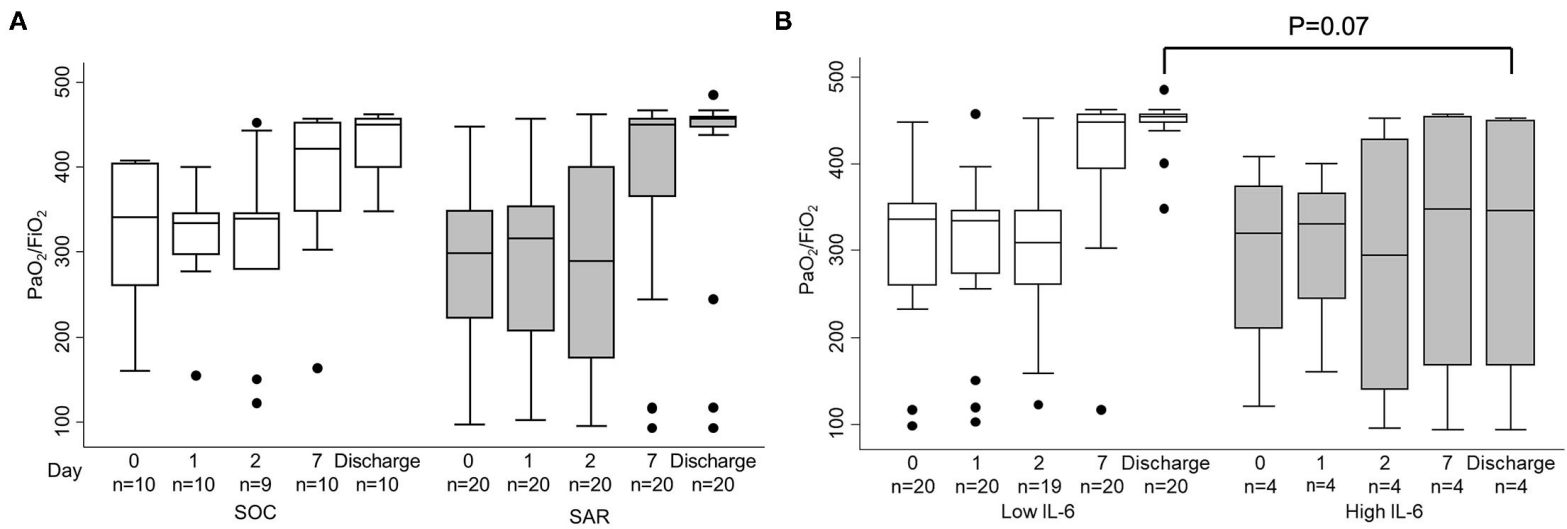
Evolution of partial pressure of arterial oxygen/fraction of inspired oxygen (PaO2/FiO2) throughout study visits. Patients are grouped depending on **(A)** allocated interventions: standard care (SC) or sarilumab (SAR) and **(B)** level of serum interleukin-6 (IL-6) at randomization (cut-off for high levels ≥ 30 pg/ml). Two patients died and their last observed value was carried forward. IL-6 levels at randomization were available only in 24 patients; high IL-6 levels were observed in 3 patients from the SAR group and 1 patient from the SC group. Data are shown as interquartile ranges (p75 upper edge of the box, p25 lower edge, p50 midline) as well as the p95 (line above box) and p5 (line below). Dots represent outliers. Statistical significance was determined with the Mann–Whitney *U* test.

Evolution of partial pressure of arterial oxygen/fraction of inspired oxygen (PaO_**2**_/FiO_**2**_) throughout study visits (Figure 3A) showed no significant differences between both allocated interventions at days 1, 2, and 7 after randomization, nor at discharge. Baseline high IL-6 levels (≥ 30 pg/ml) appeared only in 3 patients allocated to SAR and 1 to SC. Along the study visits, patients with low baseline IL-6 levels (< 30 pg/ml) tended to present non-significantly higher PaO_**2**_/FiO_**2**_ than their counterparts (Figure 3B).

**Figure 4:**
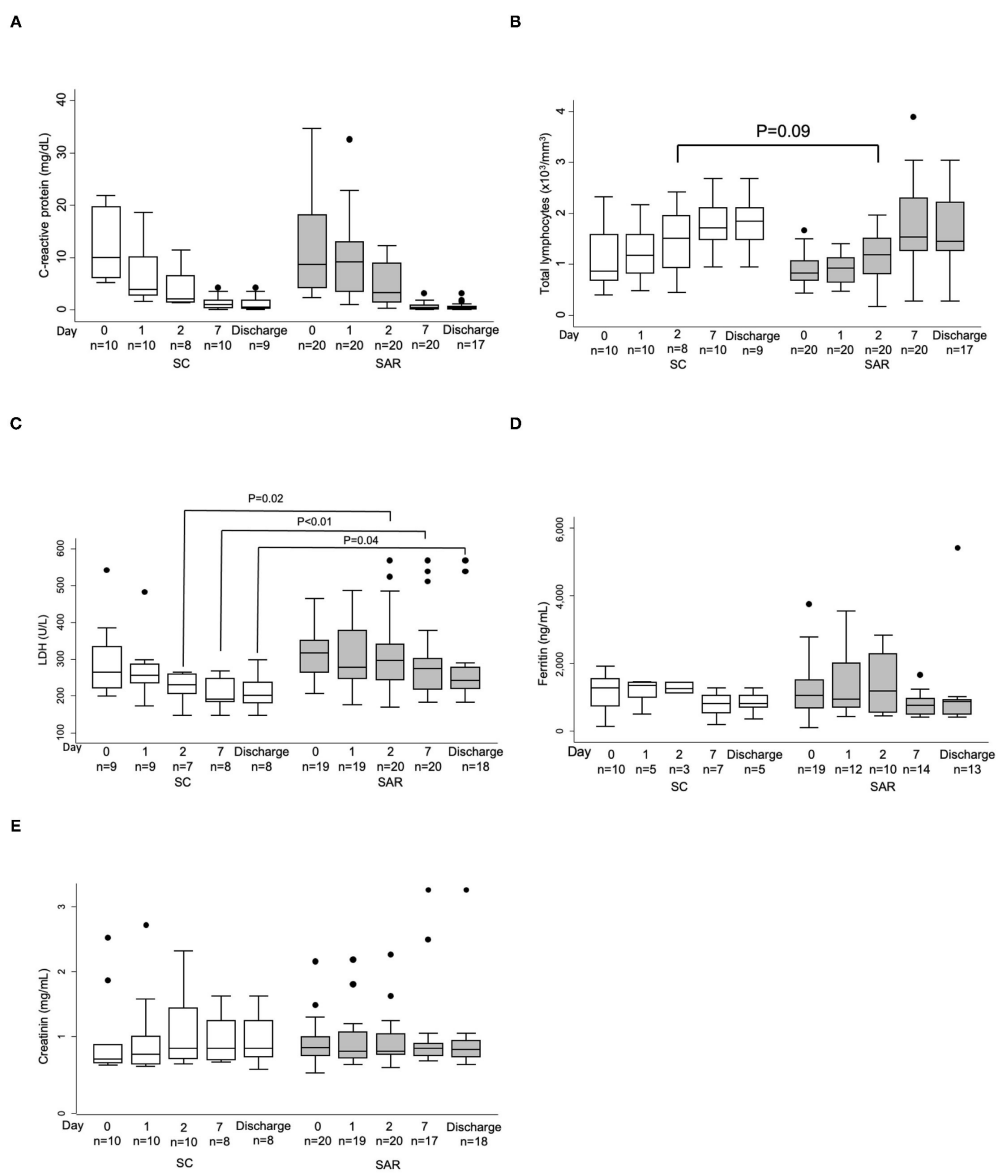
Evolution of laboratory parameters throughout study visits. **(A)** C reactive Protein; **(B)** Total lymphocyte count; **(C)** Lactate dehydrogenase; **(D)** Ferritin; **(E)** Creatinin. Patients from standard care (SC; white boxes) and sarilumab (SAR; gray boxes). Only values available at each time point is shown and results are displayed as the interquartile range (p75 upper edge of the box, p25 lower edge, p50 midline) as well as the p95 (line above box) and p5 (line below). Dots represent outliers.

Regarding surrogate inflammatory markers and laboratory parameters, no significant differences were observed between arms at baseline nor throughout the study, except for significant reductions of LDH levels after day 2 from randomization (Figure 4, and data not shown) in patients allocated to SC. The time plot decline of median CRP levels was consistent with previously reported data for sarilumab after a single 200 mg subcutaneous injection (14) with a maximum decrease on day 7, but we did not observe a steeper decrease with 400 mg SAR on days 1, 2, 4, and 5 after randomization compared to the control group (Figure 4A, and data not shown).

To avoid a possible bias, we performed a sensitivity analysis excluding the three patients that received late TCZ as SC. Baseline characteristics of these populations are shown in Supplementary Table 1, with no significant differences between arms, except for a shorter disease duration to randomization in the SAR arm (10.5 days [8–12.5]) vs. SC (18 [12–24], ***p*** = 0,013), and lower median body temperature at randomization in SC (36.3 [36.2–37]) vs. SAR (37.1 [36.6–38.1] *p* = 0.056). No significant differences were observed for primary and key secondary outcomes between allocated interventions (Table 5), confirming the results of the ITT analysis.

**Table 5:** Clinical outcomes in the sensitivity analysis population.

	**Median (IQR)**				
**Outcomes**	**Sarilumab + SC (*n* = 20)**	**SC (*n* = 7)**	**Hazard ratio (SE)**	**Log hazard ratio**	***P*** **Value**
				**(Log SE)**	
**Primary outcomes**					
Median change in clinical status (7-category ordinal scale^a^) at day 7,	2 (0–3)	3 (0–3)	-	-	0.36
Mean change in clinical status at day 7, (SD)	1.45 (1.93)	2.14 (1.46)	-	-	0.40
30-day mortality, n (%)^b^	2 (10)	0	-	15.01 (22.60)	0.54
Duration of hospitalization, days from randomization^c^	7 (6–11)	5 (4-12)	0.54 (0.25)	-	0.12
**Secondary outcomes**					
Median change in clinical status at day 14	3 (2-3)	3 (3)	-	-	0.45
Time to become afebrile for a minimum of 48 h, days^d^	3 (3-6)	15 (8-22)	5.27 (5.70)	-	0.042
Progression to NIMV, n (%)	4 (20)	0 (0)	-	16.30 (-)	0.27
Progression to IMV, n (%)	3 (15)	0 (0)	-	15.01 (22.60)	0.5
Time to supplemental oxygen withdrawal, days from randomization	6 (4-15)	3 (2-8)	0.58 (0.28)	-	0.091

*IMV, invasive mechanical ventilation; IQR, interquartile ranges; NIMV, Noninvasive mechanical ventilation; ES, standard deviation; SC, Standard care; SE, Standard error*.

a *Scale range: 1 = death to 7 = non hospitalized*.

b *One patient in the SC arm was lost to follow-up after discharging alive at day 13*.

c *Accounting for survival status by treating patients who died as having a 30-day hospital stay*.

d *Only 2 patients in the SC arm and 11 in the SAR+SC arm were febrile at randomization*.

Safety outcomes are reported in Table 6. Five SAE occurred in 4 patients allocated to SAR: 2 secondary respiratory bacterial infections by *Achromobacter xylosoxidans* and *Staphylococcus aureus*, 1 respiratory failure, and 2 fatal cases with failure of 2 organs (lung and kidney). The rate of AE of special interest was similar in SAR (50%) and SC (40%) (Table 6). Only one transient Grade III neutropenia on the SAR arm was considered as treatment related.

**Table 6:** Safety outcomes.

	**N**°**(%)**	
**Outcomes**	**Sarilumab** **+** **SC (*****n*** **=** **20)**	**SC (*****n*** **=** **10)**
**Any adverse event of special interest**		
Number of patients	10 (50)	4 (40)
Number of events, n	11	4
Neutropenia Grade IV	0	0
Increased liver enzymes^a^	5 (25)	3 (30)
Steroid-induced hyperglycemia	4 (20)	1 (10)
Invasive bacterial or fungal infection	2 (10)	0
**Serious adverse event**		
Number of patients	4 (20)	0 (0)
Number of events, n^b^	5	0
Secondary bacterial infection^c^	2 (10)	0
One organ (lung) failure	1 (5)	0
Two organ (lung/kidney) failure	2 (10)	0

*SC, Standard care*.

a *Increase in liver enzymes indicates an increase in serum levels of alanine and aspartate aminotransferases more than three times the upper limit of normal*.

b *One patient with respiratory and kidney failure under invasive mechanical ventilation also presented a respiratory bacterial infection by Achromobacter xylosoxidans*.

c *Refers to the same infection episodes described as AEs of special interest*.

### Other Outcomes

No additional deaths or serious infections were recorded by 90 days in any of the allocated arms.

## Discussion

This pragmatic open pilot RCT in hospitalized patients with moderate-to-severe COVID-19 has failed to demonstrate the benefit of adding subcutaneous SAR to the SC for preventing high oxygen requirements, invasive ventilation, or death. Additionally, serious adverse events also occurred in the intervention arm, although no definite relationship with SAR could be demonstrated.

Limited evidence based on case series (18–20) and observational studies (21–23) suggested that SAR off-label use was safe and might be beneficial in the treatment of COVID-19 infection (24). However, a systematic review and meta-analysis that included five prospective studies exploring outcomes in 389 patients who received SAR revealed that data are insufficient to establish conclusions about efficacy (25).

One retrospective case series study explored SAR in subcutaneous administration in severe and critical COVID-19 (18), suggesting a clinical benefit through early intervention before high levels of surrogate hyperinflammatory markers such as CRP or IL-6 become irresponsive. In the same way, an early observational case–control study in Italy reported survival advantage with the use of intravenous SAR when initiated in severe hyper-inflamed COVID-19 patients with a PaO2/FiO2 ≥ 100 mmHg, suggesting a potential therapeutic window of opportunity (26).

Restricted to critically ill patients, a metanalysis conducted by the international, adaptive platform trial REMAP-CAP has revealed beneficial effects of TCZ and SAR in-hospital mortality and prolonged organ support-free days in ICU (27). Those results were not validated in the first SAR RCT published in COVID-19, comparing intravenous SAR with SC in severe and critical patients (28). Neither the primary endpoint of time to improve clinical status 2 or more points on a 7-category ordinal scale nor the survival rate at day 29 showed the superiority of SAR over placebo, although a trend toward reduced mortality was observed in critically ill patients. Similar results have been reported in an early U.S. phase II/III trial, available in a non–peer-review publication (29), but the authors suggest that on patients with IMV, concomitant corticosteroids could increase the benefit of SAR.

In this regard, a recent prospective meta-analysis involving 10,930 hospitalized patients in 27 randomized trials concluded that the use of IL-6 antagonists, TCZ and SAR, was associated with a reduction in 28-day all-cause mortality, compared with SC or placebo, but this benefit was only found with concomitant administration of corticosteroids (17). SAR, mostly in the intravenous route, was assessed in nine trials, including the study reported herein, allocating 2,073 patients to SAR and 753 patients to usual care or placebo. Notably, the results were stronger for TCZ and in non–IMV-treated participants, maybe as more patients in the SAR group were under IMV and less number of patients received corticosteroids at randomization, compared to TCZ (17).

To date, no results from RCT are available supporting the use of SAR in early stages such as moderate-to-severe COVID-19. Among clinical trials planned or initiated in those stages, only two are exploring SAR in subcutaneous administration (NCT04359901, EudraCT: 2020-001531-27) and their results are awaited.

Regarding our pilot study, we outline the limitations and propose explanations for several issues behind our results.

First, a limited sample size, especially on the SC arm with no events in key endpoints such as mortality at day 30 and the need for IMV or SAE, complicated the estimation of the effect size of the intervention.

Second, assigned treatment groups were not well-balanced with several data that point to higher baseline severity in SAR arm patients. Patients on SAR were randomized earlier after disease onset compared to SC participants, suggesting a more advanced or poor prognostic disease leading to meeting the inclusion criteria in a shorter time. A higher proportion was male, had a fever, needed high-flow oxygen requirements including NIMV, or presented larger radiological lung involvement compared to SC patients. In this regard, SAR has been associated with faster recovery than SC in a subset of patients showing minor lung consolidation at baseline (23). The limited sample size prevented us from performing stratified analysis and adjustments in the multivariable models could not include all the baseline potential confounding factors.

Third, unknown comorbidities led to randomize patients with a low probability of survival. Two deaths occurred in two 72-year-old patients with previous Grade III CKD and NIMV at randomization. In addition, one of them suffered from a previous chronic thromboembolic disease with right heart failure and the other one from a serious sleep apnea hypopnea syndrome, both discovered after randomization when further information was gathered.

Fourth, the trial was not blinded and might have influenced clinical decision-making. Likely, this can explain the introduction of TCZ after randomization in some SC assigned patients to prevent further deterioration, since in usual care clinicians were used to indicate off-label TCZ following the AEMPs criteria, quite similar to ours in the trial. Nonetheless, excluding those patients from the SC arm in the sensitivity analysis did not alter the results obtained in the ITT population.

Fifth, low baseline IL-6 levels in most SAR assigned patients might have halted the potential beneficial effect of IL-6 blockade. In our experience, besides prognostic information, IL-6 levels >30 pg/ml can also predict the response to IL-6R blockade (12). However, when the protocol was designed in March 2020, we were not aware of its discriminative value for therapeutic response. Thus, we did not include this threshold as a mandatory inclusion criterion. In our previous study, no benefit of TCZ was observed in severe or critical patients with low IL-6 levels (12), and similar findings have been reported with a baseline CRP cut-off of 15 mg/dL in patients requiring ICU support (30). On the other hand, the complex biology of IL-6 and the potential dysregulation of their activities in the context of SARS-CoV2 infection should be considered to understand the effect of IL-6 blockade across different disease outcomes (31). The use of IL-6 as a biomarker of disease severity does not identify IL-6 as a unique contributor to the distinct severe manifestations in COVID-19. In a systematic review and meta-analysis of COVID-19 studies, the estimated pooled mean for IL-6 concentrations in patients with severe and critical COVID-19 was 36.7 pg/ml (95% CI 21.6–62.3 pg/ml), far lower than those reported for IL-6 and other cytokines in patients with unrelated COVID-19 ARDS, sepsis, and CAR T cell-induced cytokine released syndrome (32). This distinct inflammatory profile prompted questioning the role of a cytokine storm in COVID-19-induced organ dysfunction and considering alternative models of organ failure (32).

Sixth, the limitations of using the initial ordinal scales for primary outcomes, endorsed by the WHO early in the pandemic, has been widely recognized (33). The question remains if a threshold level of respiratory support can guide the appropriate initiation of IL-6 inhibitors.

Seventh, the widespread use of glucocorticoids on both arms (85% SAR vs. 80% SC patients) might have affected the results. Corticosteroids were commonly used in our center from the first outbreak but became the rule after the publication of their beneficial effects in the RECOVERY trial (34) and the WHO REACT metanalysis (35). However, after adjusting for a cumulative dose of glucocorticoids at day 7 after randomization, no significant differences were detected between treatment groups in the primary outcome of change in clinical status at day 7. Additionally, a cumulative dose of corticosteroids before and after randomization was similar in both arms. Concerning the simultaneous use with SAR, robust evidence has been accumulated for a greater effect of IL-6R inhibitors in concomitant use with corticosteroids (17, 27, 36). In fact, in our study, 2 out of 3 patients progressing to IMV, early recruited and allocated to SAR, were not receiving corticosteroids at randomization day and were prescribed later, followed by one patient survival.

Lately, some concerns have arisen about the subcutaneous formulation and dosage in severe patients. In line with pharmacodynamic data provided for intravenous SAR in severe and critical COVID (28, 29), with a reported rebound of CRP after declining for 7 days, a single 400 mg dose of subcutaneous sarilumab could have been sub-therapeutic. The similar time plot decline of median CRP levels in both arms in our study does not support the use of the subcutaneous route, at least with a single 400 mg dose.

## Conclusion

In our study of hospitalized patients with moderate-to-severe COVID who are not invasively ventilated at baseline, subcutaneous sarilumab added to standard care showed no additional benefit for preventing noninvasive and invasive ventilation or death by 30 days, early improvement of clinical status, or reducing hospital stay. Findings of this pilot study do not exclude a potential effect of sarilumab in moderate-to-severe COVID-19 and suggest that further blinded randomized phase III trials should be adequately powered with primary endpoint accuracy, testing higher or repeated doses, and selecting the population based on high baseline IL-6 levels. Questions remain open on subcutaneous administration and the appropriate time of intervention pending the results of more powered ongoing RCT.

## Data Availability Statement

RG-V, FA-S, and IG-A have full access to all the data in the study and take responsibility for the integrity of the data and the accuracy of the data analysis. The raw data supporting the conclusions of this article will be made available by the authors, without undue reservation.

## Ethics Statement

The studies involving human participants were reviewed and approved by Research Ethics Committee of the Hospital Universitario de la Princesa, IIS-Princesa, Madrid, Spain. Written informed consent for participation was not required for this study in accordance with the national legislation and the institutional requirements. All patients or their legal representatives provided oral informed consent according to the exceptional regulation applicable for COVID19 studies.

## Author Contributions

RG-V, IG-A, and FA-S contributed to the conception or design of the work. RG-V and SR-G contributed to the drafting of the manuscript. IG-A contributed to the statistical analysis. RG-V supervision. All authors contributed toward the acquisition, analysis, or interpretation of data, critical revision of the manuscript for important intellectual content, review and approval of the final version of the manuscript.

## Funding

This work was supported by a grant (IIS SGZ-2020-13059) to RG-V from Sanofi Spain, which also provided experimental medication (sarilumab, Kevzara^®^). Sanofi had no role in the design and conduct of the study, acquisition, management, analysis, and interpretation of the data, preparation or writing the manuscript.

## Sarcovid Trial Investigators Group

José Curbelo, Division of Infectious Diseases, Internal Medicine Service; Miguel Martinez Marín, Division of Infectious Diseases, Internal Medicine Service; Natalia Villalba, Emergency Service; Andrés von Wernitz, Emergency Service, Pedro Landete, Pneumology Service; Irene Llorente Cubas, Rheumatology Service, Eva Tomero Muriel, Rheumatology Service, Juan Pablo Baldivieso, Rheumatology Service, Esther Ramírez Herráiz, Hospital Pharmacy Service, Natalia Pascual Gómez, Laboratory Service and Nelly D Zurita, Microbiology Service, Hospital Universitario La Princesa, Instituto de Investigación Sanitaria Princesa (IIS-Princesa), Madrid, Spain.

None of whom was compensated for his or her contributions.

## Conflict of Interest

RG-V reported receiving educational grants support from Lilly, Janssen, Pfizer, Roche, Sanofi, honoraria for presentations for Lilly, Sanofi, advisory boards for Lilly, Pfizer, Sanofi, nonfinancial support from Lilly, Pfizer, and Sanofi, all outside the present work. IG-A reported Roche provided him data for research, honoraria for presentations for Lilly, Roche, Sanofi, advisory boards for Lilly, Sanofi, non-financial support from Abbvie, BMS, MSD, Novartis, Pfizer and Roche, outside the present work. The remaining authors declare that the research was conducted in the absence of any commercial or financial relationships that could be construed as a potential conflict of interest.

## Publisher's Note

All claims expressed in this article are solely those of the authors and do not necessarily represent those of their affiliated organizations, or those of the publisher, the editors and the reviewers. Any product that may be evaluated in this article, or claim that may be made by its manufacturer, is not guaranteed or endorsed by the publisher.

## References

[1] Moore JP Offit PA. SARS-CoV-2 vaccines and the growing threat of viral variants. JAMA. (2021) 325:821–2. 10.1001/jama.2021.1114 33507218

[2] WHO-Solidarity-Trial-Consortium Pan H Peto R Henao-Restrepo AM Preziosi MP Sathiyamoorthy V. Repurposed antiviral drugs for covid-19 - interim WHO solidarity trial results. N Engl J Med. (2021) 384:497–511. 10.1056/NEJMoa2023184 33264556 PMC7727327

[3] Siddiqi HK Mehra MR. COVID-19 illness in native and immunosuppressed states: a clinical-therapeutic staging proposal. J Heart Lung Transplant. (2020) 39:405–7. 10.1016/j.healun.2020.03.012 32362390 PMC7118652

[4] Mehta P McAuley DF Brown M Sanchez E Tattersall RS Manson JJ. COVID-19: consider cytokine storm syndromes and immunosuppression. Lancet. (2020) 395:1033–4. 10.1016/S0140-6736(20)30628-0 32192578 PMC7270045

[5] Zhao M Lu J Tang Y Dai Y Zhou J Wu Y. Tocilizumab for treating COVID-19: a systemic review and meta-analysis of retrospective studies. Eur J Clin Pharmacol. (2021) 77:311–9. 10.1007/s00228-020-03017-5 33051695 PMC7553373

[6] Lan SH Lai CC Huang HT Chang SP Lu LC Hsueh PR. Tocilizumab for severe COVID-19: a systematic review and meta-analysis. Int J Antimicrob Agents. (2020) 56:106103. 10.1016/j.ijantimicag.2020.106103 32712333 PMC7377685

[7] Gupta S Wang W Hayek SS Chan L Mathews KS Melamed ML. Association between early treatment with tocilizumab and mortality among critically ill patients with COVID-19. JAMA Internal Med. (2021) 181:41–51. 10.1001/jamainternmed.2020.6252 33080002 PMC7577201

[8] Le RQ Li L Yuan W Shord SS Nie L Habtemariam BA. FDA Approval summary: tocilizumab for treatment of chimeric antigen receptor T cell-induced severe or life-threatening cytokine release syndrome. Oncologist. (2018) 23:943–7. 10.1634/theoncologist.2018-0028 29622697 PMC6156173

[9] Ruan Q Yang K Wang W Jiang L Song J. Clinical predictors of mortality due to COVID-19 based on an analysis of data of 150 patients from Wuhan, China. Intensive Care Med. (2020) 46:846–8. 10.1007/s00134-020-05991-x 32125452 PMC7080116

[10] Coomes EA Haghbayan H. Interleukin-6 in Covid-19: A systematic review and meta-analysis. Rev Med Virol. (2020) 2020:e2141. 10.1101/2020.03.30.20048058 PMC7460877 32845568

[11] Tjendra Y Al Mana AF Espejo AP Akgun Y Millan NC Gomez-Fernandez C. Predicting disease severity and outcome in COVID-19 patients: a review of multiple biomarkers. Arch Pathol Lab Med. (2020) 144:1465–74. 10.5858/arpa.2020-0471-SA 32818235

[12] Galvan-Roman JM Rodriguez-Garcia SC Roy-Vallejo E Marcos-Jimenez A Sanchez-Alonso S Fernandez-Diaz C. IL-6 serum levels predict severity and response to tocilizumab in COVID-19: An observational study. J Allergy Clin Immunol. (2021) 147:72–80 e8. 33010257 10.1016/j.jaci.2020.09.018 PMC7525244

[13] Sarilumab. Summary of Product Characteristics. Available online at: https://www.ema.europa.eu/en/documents/product-information/kevzara-epar-product-information_en.pdf (accessed January 26, 2022).

[14] Ishii T Sato Y Munakata Y Kajiwara M Takahashi Y Anwar F. Pharmacodynamic effect and safety of single-dose sarilumab sc or tocilizumab iv or sc in patients with rheumatoid arthritis (RA). Ann Rheumatic Dis. (2018) 77(Suppl 2):1397–8. 10.1136/annrheumdis-2018-eular.137528850992

[15] Garcia-Vicuna R Abad-Santos F Gonzalez-Alvaro I Ramos-Lima F Sanz JS. Subcutaneous Sarilumab in hospitalised patients with moderate-severe COVID-19 infection compared to the standard of care (SARCOVID): a structured summary of a study protocol for a randomised controlled trial. Trials. (2020) 21:772. 10.1186/s13063-020-04588-5 32907638 PMC7480631

[16] WHO R&D Blueprint. Novel Coronavirus COVID-19 Therapeutic Trial Synopsis. (2020). Available online at: https://www.who.int/publications/i/item/covid-19-therapeutic-trial-synopsis (accessed January 26, 2022).

[17] WHO REACT Working Group Shankar-Hari M Vale CL Godolphin PJ Fisher D Higgins JPT. Association between administration of il-6 antagonists and mortality among patients hospitalized for COVID-19: a meta-analysis. JAMA. (2021) 326:499–518. 10.1001/jama.2021.11330 34228774 PMC8261689

[18] Montesarchio V Parrela R Iommelli C Bianco A Manzillo E Fraganza F. Outcomes and biomarker analyses among patients with COVID-19 treated with interleukin 6 (IL-6) receptor antagonist sarilumab at a single institution in Italy. J Immunother Cancer. (2020) 8:e001089. 10.1136/jitc-2020-001089 32784217 PMC7418768

[19] Benucci M Giannasi G Cecchini P Gobbi FL Damiani A Grossi V. COVID-19 pneumonia treated with Sarilumab: a clinical series of eight patients. J Med Virol. (2020) 92:2368–70. 10.1002/jmv.26062 32472703 PMC7300861

[20] Corominas H Castellvi I Diaz-Torne C Matas L de la Rosa D Mangues MA. Sarilumab (IL-6R antagonist) in critically ill patients with cytokine release syndrome by SARS-CoV2. Medicine (Baltimore). (2021) 100:e25923. 10.1097/MD.0000000000025923 34106658 PMC8133253

[21] Sinha P Mostaghim A Bielick CG McLaughlin A Hamer DH Wetzler LM. Early administration of interleukin-6 inhibitors for patients with severe COVID-19 disease is associated with decreased intubation, reduced mortality, and increased discharge. Int J Infect Dis. (2020) 99:28–33. 10.1016/j.ijid.2020.07.023 32721528 PMC7591937

[22] Gremese E Cingolani A Bosello SL Alivernini S Tolusso B Perniola S. Sarilumab use in severe SARS-CoV-2 pneumonia. EClinicalMedicine. (2020) 27:100553. 10.1016/j.eclinm.2020.100553 33043284 PMC7531933

[23] Della-Torre E Campochiaro C Cavalli G De Luca G Napolitano A La Marca S. Interleukin-6 blockade with sarilumab in severe COVID-19 pneumonia with systemic hyperinflammation: an open-label cohort study. Ann Rheum Dis. (2020) 79:1277–85. 10.1136/annrheumdis-2020-218122 32620597 PMC7509526

[24] Khiali S Rezagholizadeh A Entezari-Maleki T. A comprehensive review on sarilumab in COVID-19. Expert Opin Biol Ther. (2020) 2020:1−12. 10.1080/14712598.2021.1847269 33161757

[25] Khan FA Stewart I Fabbri L Moss S Robinson K Smyth AR. Systematic review and meta-analysis of anakinra, sarilumab, siltuximab and tocilizumab for COVID-19. Thorax. (2021) 76:907–19. 10.1101/2020.04.23.20076612 33579777

[26] Della-Torre E Lanzillotta M Campochiaro C Cavalli G De Luca G Tomelleri A. Respiratory impairment predicts response to IL-1 and IL-6 blockade in COVID-19 patients with severe pneumonia and hyper-inflammation. Front Immunol. (2021) 12:675678. 10.3389/fimmu.2021.675678 33995419 PMC8117339

[27] REMAP-CAP-Investigators Gordon AC Mouncey PR Al-Beidh F Rowan KM Nichol AD. Interleukin-6 receptor antagonists in critically Ill patients with covid-19. N Engl J Med. (2021) 384:1491–502. 10.1056/NEJMoa2100433 33631065 PMC7953461

[28] Lescure FX Honda H Fowler RA Lazar JS Shi G Wung P. Sarilumab in patients admitted to hospital with severe or critical COVID-19: a randomised, double-blind, placebo-controlled, phase 3 trial. Lancet Respir Med. (2021) 9:522–32. 10.1016/S2213-2600(21)00099-0 33676590 PMC8078879

[29] Sivapalasingam S Lederer DJ Bhore R Hajizadeh N Criner G Hosain R. A randomized placebo-controlled trial of sarilumab in hospitalized patients with Covid-19. medRxiv. (2021) 2021:2021.05.13.21256973. 10.1101/2021.05.13.21256973

[30] Biran N Ip A Ahn J Go RC Wang S Mathura S. Tocilizumab among patients with COVID-19 in the intensive care unit: a multicentre observational study. Lancet Rheumatol. (2020) 2:e603–e12. 10.1016/S2665-9913(20)30277-0 32838323 PMC7428303

[31] Jones SA Hunter CA. Is IL-6 a key cytokine target for therapy in COVID-19? Nat Rev Immunol. (2021) 21:337–9. 10.1038/s41577-021-00553-8 33850327 PMC8043092

[32] Leisman DE Ronner L Pinotti R Taylor MD Sinha P Calfee CS. Cytokine elevation in severe and critical COVID-19: a rapid systematic review, meta-analysis, and comparison with other inflammatory syndromes. Lancet Respir Med. (2020) 8:1233–44. 10.1016/S2213-2600(20)30404-5 33075298 PMC7567529

[33] McCreary EK Angus DC. Efficacy of remdesivir in COVID-19. JAMA. (2020) 324:1041–2. 10.1001/jama.2020.16337 32821934

[34] REMAP-CAP Group Horby P Lim WS Emberson JR Mafham M Bell JL. Dexamethasone in hospitalized patients with Covid-19. N Engl J Med. (2021) 384:693–704. 10.1056/NEJMoa2021436 32678530 PMC7383595

[35] WHO REACT Working Group. Association between administration of systemic corticosteroids and mortality among critically Ill patients with COVID-19: a meta-analysis. JAMA. (2020) 324:1330–41. 10.1001/jama.2020.17023 32876694 PMC7489434

[36] RECOVERY Collaborative Group. Tocilizumab in patients admitted to hospital with COVID-19 (RECOVERY): a randomised, controlled, open-label, platform trial. Lancet. (2021). 397:1637–45. 10.1016/S0140-6736(21)00676-0 33933206 PMC8084355

