# Supplementary material for: Subcutaneous IL-6 Inhibitor Sarilumab vs. Standard Care in Hospitalized Patients With Moderate-To-Severe COVID-19: An Open Label Randomized Clinical Trial
Source: Front Med (Lausanne). 2022 Feb 23;9:819621. doi: 10.3389/fmed.2022.819621 (PMC8904894; doi:10.3389/fmed.2022.819621)
Supplement: Supplementary file 1 [file Data_Sheet_1.docx]

***Supplementary Material***

**Supplementary Table 1**

**Supplementary Figure 1**

**Supplementary Table S1. Sensitivity analysis. Baseline characteristics of the study population**

|  | **n (%)** | |
| --- | --- | --- |
|  | **Sarilumab + SC (n=20)** | **SC (n=7)** |
| Age; median (IQR) | 61.5 (50.5 - 72) | 70 (60 -74) |
| Female gender; n (%) | 5 (25) | 4 (57) |
| Ethnicity, Race, n (%) |  |  |
| Caucasian | 10 (50) | 3 (43) |
| Latin-american | 10 (50) | 4 (57) |
| Coexisting Disorders, n (%) | 14 /70) | 5 (71) |
| Hypertension; n (%) | 8 (40) | 5 (71) |
| Diabetes mellitus; n (%) | 3 (15) | 2 (29) |
| Obesity; n (%) | 2 (10) | 1 (14) |
| History of Malignancy | 2 (10) | 0 |
| COPD; n (%) | 1 (5) | 1 (14) |
| Stage III Chronic kidney disease | 2 (10) | 2 (29) |
| Coronary artery disease | 3 (15) | 0 |
| Median days from symptom onset to randomization (IQR) | 10.5 (8 - 12.5) | 18 (12 - 24) |
| Median days from admission to randomization (IQR) | 2 (1 -4) | 4 (2 - 7) |
| Median body temperature at randomization (IQR), °C | 37.1 (36.6 - 38.1) | 36.3 (36.2 - 37.0) |
| Fever >37,5 °C, n (%) | 9 (45) | 0 |
| Oxygen support at randomization (7-point functional ordinal scale) n (%) |  |  |
| 5. No supplemental oxygen therapy | 4 (20) | 0 |
| 4. Supplemental low flow oxygen therapy ^a^ | 12 (60) | 7 (100) |
| 3. Supplemental high flow oxygen therapy or NIMV ^b^ | 4 (20) | 0 |
| PaO2/FiO2; median (IQR) | 298 (223 - 348) | 264 (233 - 350) |
| **Additional treatment during hospitalization** |  |  |
| Hydroxychloroquine use; n (%) | 4 (20) | 2 (29) |
| LPVR use; n (%) | 4 (20) | 1 (14) |
| Azithromycin use; n (%) | 12 (60) | 4 (57) |
| Glucocorticoisteroid use ^c^; n (%) | 17 (85) | 6 (86) |
| Methylprednisolone bolus at randomization | 14 (70) | 3 (43) |
| **Laboratory Values (median, IQR)** |  |  |
| White Blood Count (cells/mm^3^) | 7070 (4975 - 12310) | 9910 (4650 - 10460) |
| Lymphocyte Count (cells/mm^3^) | 825 (680 - 1070) | 1340 (820 - 1780) |
| Creatinine. mg/dl | 0.825 (0.705 - 0.99) | 0.6 (0.59 - 1.86) |
| Bilirubin. mg/dl | 0.38 (0.31 - 0.52) | 0.42 (0.25 - 0.51) |
| AST. U/L | 40 (26.5 - 53) | 33 (25 - 99) |
| ALT. U/L | 48 (29 - 56.5) | 51 (17 - 97) |
| GGT. U/L | 40 (29 - 118) | 68 (55 - 108) |
| LDH. U/L | 317 (263 - 350) | 285 (222 - 333) |
| **Inflammatory markers (median, IQR)** |  |  |
| serum IL-6. pg/ml (n=21) | 13.3 (7.5 - 24) | 3 (1 - 3) |
| **(Continued)** | **n (%)** | |
|  | **Sarilumab + SC (n=20)** | **SC (n=7)** |
| IL-6 ≥ 30 pg/ml , n (%) | 3/16 (18.8) | 1 /7 (14) |
| Ferritin. ng/mL | 1351 (735 - 1639) | 1048 (664 - 1511) |
| Procalcitonin ng/ml | 0.11 (0.09 - 0.18) | 0.115 (0.07 - 0.175) |
| D-dimmer. µg/ml | 0.49 (0.36 - 1.28) | 0.64 (0.48 - 1.42) |
| CRP (mg/dL); median (IQR) | 8.58 (4.16 - 18.10) | 7.35 (6.03 - 19.73) |
| **Thorax radiologic findings (x ray and/or CT scan) ^d^** |  |  |
| Alveolar pattern or ground glass opacities > 50% | 11 (55) | 3 (43) |

AST:Aspartate amino-transferase; ALT: Alanine amino- transferase; COPD: chronic obstructive pulmonary disease; CRP: C-reactive protein; CT: Computerized scan; GGT: Gamma-glutamyl transferase; IL6: interleukin 6; IMV: invasive mechanical ventilation; IQR: interquartile ranges; LDH: Lactate Dehydrogenase; LPVR: Lopinavir/Ritonavir; NIMV: Noninvasive mechanical ventilation; PaO2/FiO2: arterial oxygen tension/fraction of inspired oxygen ratio ;SC: Standard care.

**^a^** O2 flow ≤ 15l/min e.g. by face mask, nasal cannula (NC)

**^b^** O2 flow >15l/min, e.g. by face mask, ‘High Flow’ devices (e.g. HFNC), CPAP or NIV including BiPAP and other devices

**^c^** Corticosteroids: ≥ 30 mg Prednisone/d or equivalent; endovenous bolus of 6-Metilprednisolone 120-125 mg/d,

**^d^** All radiologic exams were assessed and reported by radiologists with pneumological expertise


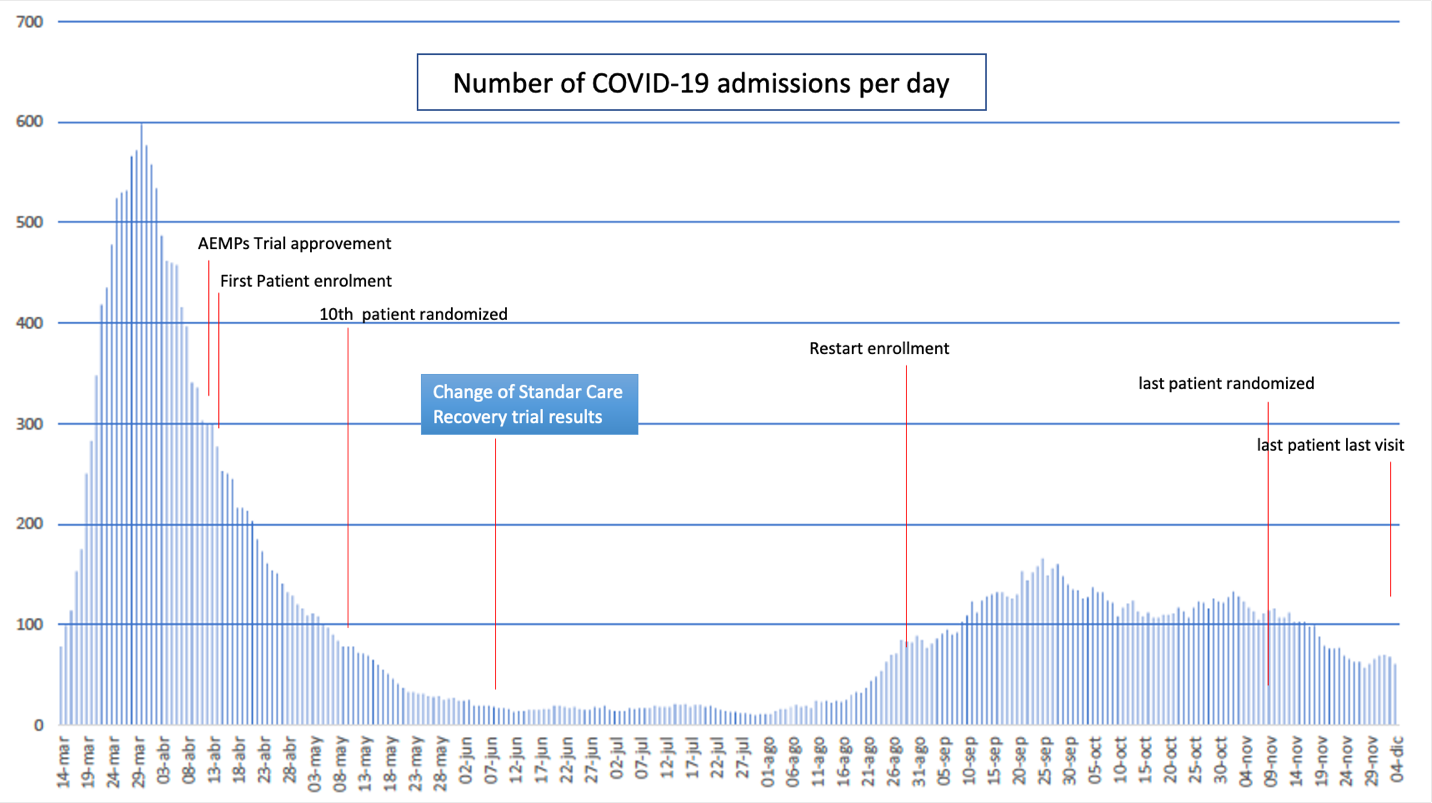


**Spplementary Figure S1. Timeline of trial recruitment.**

The graphic illustrates the number of COVID-19 admissions per day at the Hospital Universitario la Princesa throughout the trial, reflecting changes in incidental cases in the autonomous region of Madrid. Key dates are depicted, from regulatory authorities’ approval (Spanish Agency for medicinal and health products, AEMPS) to the last patient last visit.
